# The effect of anatomical variations of the sinonasal region on maxillary sinus volume and dimensions: a three-dimensional study

**DOI:** 10.1016/j.bjorl.2021.05.001

**Published:** 2021-05-18

**Authors:** Firdevs Aşantoğrol, Aykağan Coşgunarslan

**Affiliations:** a Erciyes University, Faculty of Dentistry, Department of Oral and Maxillofacial Radiology, Kayseri, Turkey; b Niğde Oral and Dental Health Center, Niğde, Turkey

**Keywords:** Sinonasal region, Anatomical variations, Maxillary sinus volume, Three-dimensional analysis

## Abstract

**Introduction:**

Anatomical variations in the sinonasal region are remarkably common and these variations may have an impact on maxillary sinus development due to their close anatomical adjacency.

**Objective:**

The aim of this study was to investigate the effect of anatomical variations of the sinonasal region on the width, height, length, and volume of the maxillary sinus.

**Methods:**

Cone beam computed tomography records of 120 patients were evaluated. Nasal septum deviation angle was measured for each patient and patients were divided into three groups as mild, moderate, and severe. Sinonasal variations such as nasal septum deviation direction, septal spur, concha bullosa, uncinate process pneumatization, middle concha hypertrophy, inferior concha hypertrophy, paradoxical middle concha and presence of septa in the maxillary sinus were registered. Cone beam computed tomography images were transferred to the SimPlant software thus right and left maxillary sinus volumes and dimensions were measured separately.

**Results:**

There was a negative and statistically significant relationship between age and left maxillary sinus width (*p* = 0.015). The relationships between gender and maxillary sinus volumes and dimensions were statistically significant (*p* < 0.05). Although there were significant relationships between the nasal septum deviation severity, middle concha hypertrophy, inferior concha hypertrophy, paradoxical middle concha, and presence of septa and maxillary sinus dimensions; there was no significant relationship between septal spur, nasal septum deviation direction, concha bullosa, uncinate process pneumatization, and maxillary sinus dimensions. No statistically significant relationship could be determined between maxillary sinus volume and anatomical variations of the sinonasal region.

**Conclusion:**

According to our findings, while certain the variations were found to affect the sinus dimensions; none of the variations was found to be related to the maxillary sinus volume.

## Highlights

• The maxillary sinus and the nasal structures have a close anatomical relationship.
• Anatomical variations are very common in the sinonasal region.
• Nasal septal deviation during the developmental period may result in facial asymmetry.
• The relationship between concha hypertrophy and sinus height was significant.
• A significant relationship was found between paradoxical concha and sinus width.

## Introduction

The pyramid-shaped maxillary sinus is the largest paranasal sinus, located just behind the anterior bone surface of the midface and surrounded by bone structures.^1^ Development of maxillary sinuses begins in the prenatal period. Maxillary sinus volume at birth varies between 6 and 8 cm cubed (cm^3^). During the postnatal period, the phase from birth to three years old and the phase between the ages of 7 and 12 are the two fastest development phases of the maxillary sinus.^2^ Maxillary sinus continues to develop between the ages of 12 and 15 and reach its adult size at age of 15.^3^

The maxillary sinus and the nasal structures have a close anatomical relationship with each other. The lateral wall of the nasal cavity forms the medial wall of the maxillary sinus. Quite complex structures and many anatomical variations can be seen in the sinonasal region. Nasal septum deviation (NSD) is one of the most common variations. It has been reported in the literature that septal cartilage has an important role in the facial growth,^4^, ^5^ and that septal deviation during the developmental period may result in facial asymmetry.^6^

In the current literature, there are studies investigating the effect of NSD, concha bullosa (CB), and the presence of septa on maxillary sinus volume.^7^, ^8^, ^9^, ^10^, ^11^, ^12^, ^13^, ^14^, ^15^ However, to the best of our knowledge, there are no published studies assessing the relationship between sinus dimensions and volumes and other anatomical variations such as septal spur (Sspur), uncinate process pneumatization (UPP), middle concha hypertrophy (MCH), inferior concha hypertrophy (ICH), paradoxical middle concha (PMC). The present study is the first study to examine the effect of all anatomical variations of the sinonasal region on maxillary sinus volume (MSV) and dimensions.

## Methods

This retrospective study was approved by the Clinical Research Ethics Committee (protocol nº 2020/20). CBCT images were obtained from 120 patients assisted at the Faculty of Dentistry Department of Oral and Maxillofacial Radiology for various reasons between the dates of April 2017 and June 2019. Exclusion criteria were listed as, individuals with incomplete maxillary sinus growth and development, individuals with skeletal deformities in the midface region, individuals with a history of sinonasal region surgery or trauma history, individuals with loss of teeth in the posterior maxillary region, individuals with maxillary impacted teeth, and individuals with benign/malignant tumors affecting the sinonasal region.

All the CBCT images were obtained with the New Tom 5 G CBCT device (FP, Quantitative Radiology, Verona, Italy). All images were recorded at 110 kV and 3–5 mA, with 0.25 mm voxel size, 0.25 mm slice thickness, 18 × 16 field of size, and had a typical exposure time of 5.4 s. CBCT images were analyzed in a dark room, with a Dell Precision T5400 workstation (Dell, Round Rock, TX, USA), using NNT software (NNT software, version 3.0; NewTom, Verona, Italy) and 32 inch Dell LCD with a resolution of 1280 × 1024 pixels. Afterward, the images were transferred to the SimPlant software (version 13.0: Materialize, Leuven, Belgium) in DICOM format for measurements.

The examination of the images and measurements were both made by two researchers separately. Randomly selected images (20% percent) were re-evaluated in 15 days for inter-observer and intra-observer reliability.

### Examination of sinonasal anatomical variations

**Figure 1:**
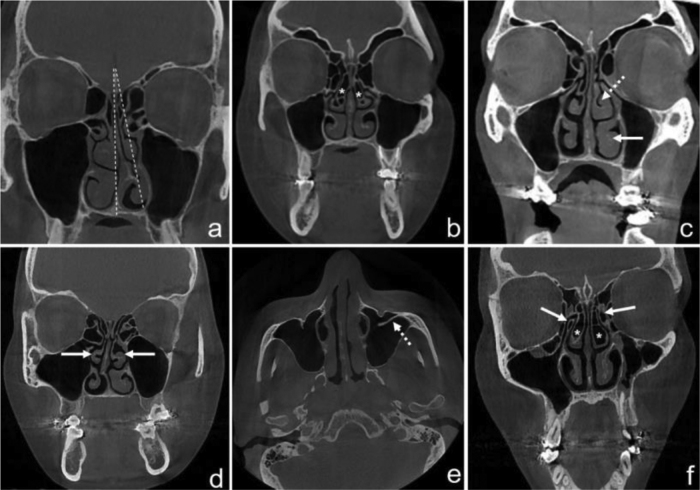
CBCT images of sinonasal anatomical variations. Septal deviation angle (a); bilateral CB (asterisk) (b); middle concha hypertrophy (MCH) (dashed arrow), inferior concha hypertrophy (ICH) (white arrow) (c); bilateral paradoxical middle concha (PMC) (white arrows) (d); presence of septa in the maxillary sinus (dashed arrow) (e); bilateral uncinate process pneumatization (UPP) (white arrows) and CB (asterisk) (f).

Multiplanar reformat images including axial, coronal, and sagittal sections were used to evaluate the nasal cavity and maxillary sinus. If any of the variations mentioned below were found, they were recorded as unilateral left-sided, unilateral right-sided, or bilateral (Fig. 1).

Severity of nasal septum deviation (NSD): NSD angle was measured between the midline which was formed by crista galli and anterior nasal spine and the most deviated point of the nasal septum in the coronal plane. Patients were then divided into 3 groups according to the classification made by Elahi et al.;^16^ G-1: mild (<9°); G-2: moderate (9°–15°); G-3: severe (>15°).

Septal spur (Sspur): it was determined as a bone protrusion of the nasal septum in coronal and axial CBCT sections.

NSD direction: NSD direction was noted as right or left according to the convexity of the septal curvature.

Concha bullosa (CB): CB was considered as any pneumatization of the middle concha, which normally does not contain air. The examination of the presence of CB was made in coronal and axial CBCT sections.

Uncinate process pneumatization (UPP): UPP was considered as any presence of air in the uncinate process on coronal CBCT sections.

Middle concha hypertrophy (MCH) and inferior concha hypertrophy (ICH): hypertrophy was considered as an enlargement of the concha, secondary to the thickening of soft-tissue and/or bone components. The presences of MCH and ICH were examined on coronal CBCT images.

Paradoxical middle concha (PMC): PMC was determined by the convexity from the lateral to medial, in contrast to the normal course of the middle concha. PMC examination was made on coronal CBCT images.

Presence of septa in the maxillary sinus: presence of completed or incomplete septa, originating from the sinus walls was assessed on the coronal and sagittal CBCT images.

### Measurements of maxillary sinus volume and dimensions

The measurements of sinus dimensions were performed by scanning sequential sections in which maximum dimensions can be measured in coronal and axial CBCT images.

**Figure 2:**
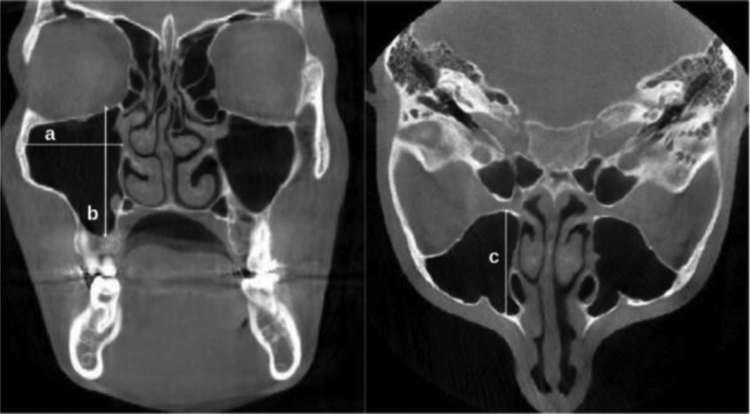
The coronal and axial CBCT images revealing the measurement of the MSW, MSH, and MSL. MSW; the longest distance from the most medial wall of the sinus to the most lateral wall of the sinus (a), MSH; the longest distance between the sinus floor and the sinus roof (b), MSL; the longest distance between the anterior wall and the posterior wall of the sinus (c).

The maxillary sinus width (MSW) was determined as the distance between the most medial and the most lateral wall of the sinus, drawn perpendicularly to the medial wall of the maxillary sinus in the coronal plane. The maxillary sinus height (MSH) was determined as the longest distance between the sinus floor and the sinus roof in the coronal plane. Additionally, the longest distance between the anterior wall and the posterior wall of the sinus, measured on axial images was recorded as the maxillary sinus length (MSL) (Fig. 2).

**Figure 3:**
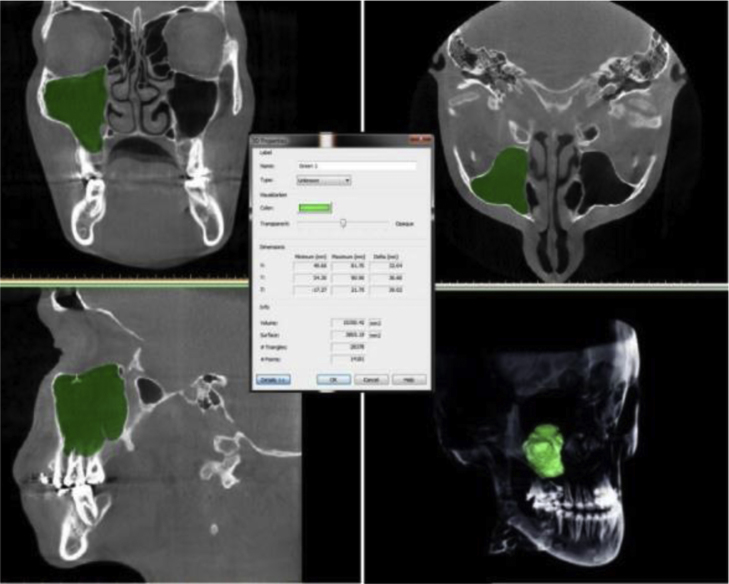
3D volume reconstruction of the maxillary sinus.

Gray scale values corresponding to Hounsfield values were adjusted at maximum voxel size in each patient for MSV measurements. The arrangement of the masks and segmentation of the sinuses were performed manually, and the connection of the maxillary sinus with the nasal cavity and other anatomical structures was erased separately in the axial, coronal, and sagittal planes. After the editing process of the masks, the volume calculation of the sinuses was carried out by the software (Fig. 3). Volume data was measured in mm^3^.

### Statistical analysis

The analysis of the data was performed using the SPSS Statistics v. 22.0 software (IBM Corp., Armonk, NY, USA). Statistical significance was set as α = 0.05. The conformity of continuous variables to normal distribution was tested with the Shapiro–Wilk test. Descriptive statistics for continuous variables were given as median (minimum–maximum) for those with no conformity to the normal distribution, mean ± standard deviation for those with conformity to the normal distribution, and frequency and related percentage values for categorical variables. Kruskal–Wallis test, Mann–Whitney *U* test, one-way variance analysis, independent sample *t*-test, and Pearson Chi-Square test were used for comparisons between groups. Bonferroni correction was used when a significant difference was obtained in more than two-group comparisons. The relationship between continuous variables was determined by the Spearman rank correlation coefficient. Intraclass correlation coefficients (ICC) were calculated to evaluate the intra-observer and inter-observer reliability.

## Results

The intra-observer reliability was evaluated and the ICC values were found between the range of 0.885 and 0.946. The ICC values for inter-observer reliability ranged between 0.934 and 0.986. 120 patients (70 female [58.3%] and 50 male [41.7%]) were included. The age of the included patients ranged between 16–44, with a mean age of 22.2. The mean right maxillary sinus volume (RMSV) value was 14.024 ± 5.32 cm^3^, while the mean left maxillary sinus volume (LMSV) was 13.184 ± 4.09 cm^3^. The mean MSW, MSH and MSL values for the right and left sides were found as 26.72 ± 4.3, 26.05 ± 4.2 mm; 36.97 ± 5.2, 36.31 ± 5.4 mm; and 37.24 ± 3.5, 36.69 ± 3.2 mm respectively.

A statistically significant and negative correlation was found between the age and the left maxillary sinus width (LMSW) (r: Spearman's rank correlation coefficient; r = −0.348) (p = 0.015). Maxillary sinus dimensions and MSV values were found higher in males compared to females. The relationships between the gender variable, and MSV and maxillary sinus dimensions were statistically significant (*p* < 0.05).

Frequencies of the variations were found as: NSD; 66.7%, Sspur; 29.1%, CB; 62.5% (14.1% right-sided, 19.2% left-sided, 29.2% bilateral), MCH; 12.5% (10.8% right-sided, 1.7% left-sided), ICH; 68.3% (22.5% right-sided, 33.3% left-sided, 12.5% bilateral), POC; 26.7% (10.9% right-sided, 2.5% left-sided, 13.3% bilateral), UPP; 17.5% (1.7% right-sided, 2.5% left-sided, 13.3% bilateral), precense of septa in the maxillary sinus; 17.5% (13.3% right-sided, 1.7% left-sided, 2.5% bilateral). There was no significant difference regarding the distribution pattern of the variations between the gender types (*p* > 0.05).

The relationship between the NSD severity and the MSV and sinus dimensions were shown in Table 1, with paired comparisons. Statistically significant differences were found between the MSW and NSD severity in both right and left sides (*p* = 0.042, *p* = 0.007). This difference was found to be resulted from the differences between the subgroups 0–1, 1–2, and 1–3 (Table 1). The effects of NSD direction, presence of Sspur, CB, and UPP were not found to be significant on the MSV and sinus dimensions (Table 2, Table 3, Table 4, Table 5). There was a statistically significant difference between the MCH and right maxillary sinus height (RMSH) variables (*p* = 0.003) (Table 6).

**Table 1:** Comparisons of the maxillary sinus volume and dimensions according to the severity of NSD.

	0 (n = 40)	1 (n = 33)	2 (n = 30)	3 (n = 17)	*p*-value
RMSW	25.82 ± 5.01	29.99 ± 4.01	24.93 ± 5.14	25.82 ± 4.25	0.042
RMSH	35.67 ± 6.46	39.80 ± 3.51	37.07 ± 6.86	34.55 ± 5.06	0.170
RMSL	36.73 ± 4.91	39.47 ± 3.16	35.54 ± 2.74	37.24 ± 2.71	0.072
RMSV	14.92 (6.88–18.95)	14.63 (11.07–23.03)	11.32 (4.18–28.51)	14.85 (5.72–18.43)	0.131
LMSW	25.50 (15.18–28.60)	29.31 (22.37–38.80)	22.65 (18.25–32.66)	25.98 (16.26–28.12)	0.007
LMSH	36.22 (21.86–44.62)	38.99 (31.74–44.80)	37.44 (22.25–43.87)	35.05 (24.60–39.06)	0.276
LMSL	35.55 ± 5.50	38.06 ± 2.79	36.34 ± 2.77	37.36 ± 2.48	0.362
LMSV	14.07 (1.40–19.25)	15.91 (10.19–20.26)	9.84 (6.46–27.49)	12.78 (4.65–17.51)	0.142

Pairwise comparisons		
Group	RMSW	LMSW
0–1	0.130	0.001
0–2	1.000	0.909
0–3	1.000	0.769
1–2	0.049	0.026
1–3	0.386	0.005
2–3	1.000	0.773

NSD, Nasal septal deviation and p < 0.05 statistically significant.

Groups: 0: absent; 1: mild; 2: moderate; 3: severe; dimensional measurements: mm; volumetric measurements: cm^3^.

**Table 2:** The correlation of the maxillary sinus volume and dimensions with Sspur.

	0 (n = 85)	1 (n = 35)	*p*-value
RMSW	26.68 ± 5.43	26.83 ± 3.83	0.928
RMSH	36.77 (19.89–47.33)	38.37 (27.06–42.99)	0.447
RMSL	37.31 ± 4.38	37.10 ± 2.49	0.866
RMSV	14.17 ± 4.96	13.69 ± 3.47	0.748
LMSW	26.46 ± 4.88	25.06 ± 4.63	0.363
LMSH	36.55 (21.86–44.80)	38.11 (24.60–41.73)	0.468
LMSL	36.53 ± 4.31	37.09 ± 2.82	0.655
LMSV	13.64 ± 5.18	12.14 ± 3.59	0.333

Sspur, Septal spur and *p* < 0.05 statistically significant.

Groups: 0: absent; 1: present; dimensional measurements: mm; volumetric measurements: cm^3^.

**Table 3:** The correlation of the maxillary sinus volume and dimensions with the direction of NSD.

	0 (n = 40)	1 (n = 40)	2 (n = 40)	*p*-value
RMSW	25.825.01	27.50 ± 5.49	26.86 ± 4.57	0.640
RMSH	35.68 ± 6.46	37.84 ± 6.60	37.41 ± 4.44	0.551
RMSL	36.73 ± 4.91	37.49 ± 2.85	37.53 ± 3.85	0.814
RMSV	14.13 ± 3.69	15.01 ± 5.53	12.95 ± 4.09	0.444
LMSW	25.50 (15.18–28.60)	27.43 (18.25–38.80)	26.54 (16.26–34.47)	0.236
LMSH	35.33 ± 6.41	36.66 ± 5.76	36.96 ± 4.46	0.682
LMSL	36.65 (23.88–44.74)	37.06 (32.43–42)	37.96 (31.43–42.61)	0.661
LMSV	12.68 ± 5.24	14.23 ± 5.32	12.58 ± 3.77	0.563

NSD, Nasal septal deviation and *p* < 0.05 statistically significant.

Groups: 0: absent; 1: right sided; 2: left sided.

**Table 4:** Correlation of the maxillary sinus volume and dimensions with CB.

	0 (n = 45)	1 (n = 17)	2 (n = 23)	3 (n = 35)	*p*-value
RMSW	26.45 ± 4.90	25.19 ± 3.21	28.78 ± 4.74	26.53 ± 5.94	0.530
RMSH	37.05 ± 5.91	35.45 ± 4.79	37.78 ± 4.64	37.12 ± 7.29	0.890
RMSL	36.83 ± 3.94	37.20 ± 2.95	38.43 ± 2.77	37.05 ± 4.95	0.796
RMSV	13.72 ± 4.20	12.67 ± 3.51	15.89 ± 5.42	13.85 ± 4.81	0.534
LMSW	25.90 ± 5.32	23.73 ± 4.23	28.68 ± 5.05	25.72 ± 3.84	0.223
LMSH	36.58 ± 5.69	34.73 ± 6.41	37.37 ± 4.18	36.08 ± 6	0.818
LMSL	36.06 ± 3.96	37.43 ± 3.61	37.45 ± 2.36	36.65 ± 4.91	0.798
LMSV	13.47 ± 4.07	11.80 ± 3.99	15.42 ± 5.88	12.11 ± 5.01	0.351

Groups: 0: absent; 1: right sided; 2: left sided; 3: bilateral.

CB, Concha bullosa and *p* < 0.05 statistically significant.

**Table 5:** Correlation of the maxillary sinus volume and dimensions with UPP.

	0 (n = 99)	3 (n = 16)	*p*-value
RMSW	26.70 ± 4.66	26.676.97	0.991
RMSH	36.16 ± 5.92	40.16 ± 4.21	0.121
RMSL	36.90 ± 4.06	38.83 ± 3.23	0.275
RMSV	13.90 ± 4.09	13.19 ± 4.32	0.701
LMSW	26.11 ± 5.07	25.82 ± 3.30	0.894
LMSH	36.31 (21.86–44.80)	38.11 (34–41.73)	0.549
LMSL	36.35 ± 3.73	39.12 ± 4.06	0.103
LMSV	13.29 ± 4.02	13.07 ± 3.60	0.901

UPP, Uncinate process pneumatization. Due to the small number of patients, Group 1 and 2 were not included in the comparison.

Groups: 0: absent; 1: right sided; 2: left sided; 3: bilateral.

**Table 6:** Correlation of the maxillary sinus volume and dimensions with MCH.

	0 (n = 105)	1 (n = 13)	*p*-value
RMSW	26.44 ± 5.27	28.43 ± 1.95	0.411
RMSH	36.49 ± 6.20	40.26 ± 1.49	0.003
RMSL	37.11 ± 4.16	38.26 ± 1.94	0.548
RMSV	13.85 ± 4.82	15.30 ± 2.59	0.513
LMSW	26.12 ± 4.98	27.79 ± 1	0.070
LMSH	36.85 (21.86–44.80)	39.06 (31.74–40.29)	0.656
LMSL	36.61 ± 4.12	38.15 ± 1.54	0.417
LMSV	13.63 (1.40–27.49)	13.01 (12.43–16.05)	0.830

MCH, Middle concha hypertrophy. Due to the small number of patients, Group 2 and 3 were not included in the comparison.

Groups: 0: absent; 1: right sided; 2: left sided; 3: bilateral.

Statistically significant differences were found between ICH and dimension measurements such as right maxillary sinus width (RMSW), RMSH, right maxillary sinus length (RMSL), left maxillary sinus height (LMSH) and left maxillary sinus length (LMSL) (*p* = 0.039, *p* = 0.042, *p* = 0.004, *p* = 0.035, and *p* = 0.006). It has been understood with paired comparisons that the difference between ICH and RMSW was resulted from the difference between subgroups 2–3 (*p* = 0.028). The difference between ICH and RMSH resulted from the differences between subgroups 0–3, 1–3 and 2–3 (*p* = 0.018, p = 0.020, *p* = 0.003). The difference between the ICH and LMSH was influenced by the differences between subgroups 0–3, and 2–3. (*p* = 0.045, *p* = 0.010) Lastly, the differences between the ICH and MSL values were influenced by the differences between subgroups 0–3, 1–3, and 2–3. (For RMSL; *p* = 0.001, *p* = 0.001, *p* = 0.001) (For LMSL; *p* = 0.002, *p* = 0.003, *p* = 0.005) (Table 7).

**Table 7:** Correlation of the maxillary sinus volume and dimensions with ICH.

	0 (n = 38)	1 (n = 27)	2(n = 40)	3 (n = 15)	*p*-value
RMSW	26.79 ± 3.76	27.21 ± 3.46	28.26 ± 5.80	21.58 ± 5.35	0.039
RMSH	38.91 (28.45–47.33)	37.77 (31.34–46.97)	38.35 (25.83–45.15)	31.36 (19.89–36.31)	0.042
RMSL	37.39 (29.77–40.31)	38.03 (34.93–40.98)	39 (31.81–46.49)	32.02 (25.70–35.73)	0.004
RMSV	14.52 (8.72–28.51)	12.98 (11.07–18.65)	14.77 (5.72–23.03)	11.77 (4.18–15.43)	0.295
LMSW	25.81 (20.39–31.45)	27.69 (24.51–32.66)	27.50 (16.26–38.80)	21.68 (15.18–29.37	0.105
LMSH	36.85 (31.11–43.87)	36.13 (31.74–44.80)	38.95 (21.86–44.62)	29.90 (22.25–38.13)	0.035
LMSL	36.70 (31.43–39.57)	37.11 (34.51–41.18)	39.10 (30.58–44.74)	30.97 (23.88–36.37)	0.006
LMSV	13.26 (8.97–27.49)	13.56 (1.40–19.35)	14.26 (4.65–20.26)	9.69 (5.67–14.33)	0.488

Pairwise comparisons					
Group	RMSW	RMSH	RMSL	LMSH	LMSL
0–1	1.000	1.000	0.919	0.959	0.683
0–2	1.000	0.740	0.202	0.086	0.151
0–3	0.156	0.018	0.001	0.045	0.002
1–2	1.000	0.680	0.318	0.272	0.178
1–3	0.133	0.020	0.001	0.062	0.003
2–3	0.028	0.003	0.001	0.010	0.005

ICH, Inferior concha hypertrophy, *p* < 0.05 statistically significant.

Groups: 0: absent; 1: right sided; 2: left sided; 3: bilateral.

A statistically significant difference was found between PMC and LMSW. This difference was resulted from the difference between subgroups 0–1 (*p* < 0.001) (Table 8). Statistically significant differences were found between the presence of septa and the MSW for both sides. (For RMSW: *p* = 0.021, for LMSW: *p* = 0.049) (Table 9).

**Table 8:** Correlation of the maxillary sinus volume and dimensions with POC.

	0 (n = 88)	1 (n = 13)	3 (n = 16)	*p*-value
RMSW	26 ± 4.84	28.82 ± 3.51	29.03 ± 6.92	0.252
RMSH	36.85 ± 6.35	38.09 ± 3.30	36.82 ± 6.45	0.912
RMSL	36.81 ± 4.11	37.38 ± 1.63	39.43 ± 4.45	0.342
RMSV	13.99 ± 4.89	13.57 ± 1.42	15.15 ± 5.17	0.827
LMSW	25.90 (15.18–31.45)	29.64 (28.69–32.66)	28.98 (16.26–38.80)	0.009
LMSH	36.58 (21.86–44.62)	38.13 (36.13–40.67)	36.85 (24.60–44.80)	0.637
LMSL	35.92 ± 4.21	38.17 ± 1.37	38.89 ± 2.83	0.153
LMSV	12.59 ± 5.08	14.59 ± 1.46	15.09 ± 5.77	0.421

Pairwise comparisons	
Group	LMSW
0–1	<0.001
0–3	0.271
1–3	0.792

POC, Paradoxic middle concha, *p* < 0.05 statistically significant. Due to the small number of patients, Group 2 were not included in the comparison.

Groups: 0: absent; 1: right sided; 2: left sided; 3: bilateral.

**Table 9:** Correlation of the maxillary sinus volume and dimensions with the presence of the maxillary sinus septa.

	0 (n = 99)	1 (n = 16)	*p*-value
RMSW	25.82 ± 4.66	30.85 ± 5.50	0.021
RMSH	35.95 ± 6.02	40.55 ± 3.63	0.078
RMSL	36.72 ± 4.07	39.43 ± 2.30	0.122
RMSV	13.53 ± 4.20	15.48 ± 2.28	0.278
LMSW	25.36 ± 4.60	29.52 ± 5.02	0.049
LMSH	37.39 (21.86–44.62)	35.59 (31.74–44.80)	0.905
LMSL	36.25 ± 4.08	38.81 ± 3.12	0.151
LMSV	12.62 ± 4.46	14.36 ± 3.27	0.366

Groups: 0: absent: 1: right sided, 2: left sided, 3: bilateral; *p* < 0.05 statistically significant. Due to the small number of patients, Group 2 and 3 could not be included in the comparison.

No significant relation was found between the evaluated anatomical variations and MSV (Table 1, Table 2, Table 3, Table 4, Table 5, Table 6, Table 7, Table 8, Table 9).

## Discussion

Anatomical variations are quite common in the sinonasal region. The effects of variations such as NSD and CB on maxillary sinusitis have been investigated adequately. However, the relationship between these variations and the development and volume of the maxillary sinus is still controversial. Different mechanisms such as brain growth and muscular traction play a role in the growth of the sinus cavities.^13^ In addition, airflow through the nasal cavities affects the development of the paranasal sinuses and the craniofacial skeleton.^15^, ^16^, ^17^ It has been thought that the interruption of the nasal airflow by anatomic variations can affect the sinus development and volume.^15^ The aim of this study was to investigate the relationship between MSW, MSH, MSL, and MSV and anatomical variations of the nasal cavity through CBCT images.

Different imaging techniques such as CBCT,^7^, ^8^, ^10^ and CT^9^, ^11^, ^12^, ^13^, ^14^, ^15^ have been used to evaluate the nasal cavity and maxillary sinuses in the literature. Conventional radiographs allow only two-dimensional evaluation of anatomical structures while CT and CBCT provide valuable information in the imaging and identification of anatomical variations in the bone structures of the paranasal sinuses. CBCT has advantages such as relatively low radiation dose, low cost, and a short time to obtain images compared to CT. CBCT usage in fields of dentistry and rhinology keeps increasing.^18^ With CBCT, it is possible to obtain very thin multiplanar-reformatted images with high resolution. Thus, it provides a better understanding of the complex anatomy of the maxillary sinus and nasal structures.^19^, ^20^

Previous studies have used different software to measure the MSV. In the present study, three-dimensional volumetric analysis along with measurements of dimension parameters such as width, height, and length of the maxillary sinus were performed as well as their relationships with sinonasal variations. Differences in methodology should be considered for possible differences when comparing findings in the literature.

MSV values and sinus dimensions can vary with age and may differ between individuals. Moreover, the right and left sinus parameters of the same individual may also differ.^21^ Studies in the current literature have reported no significant difference between the RMSV and LMSV.^7^, ^8^, ^9^, ^10^, ^12^, ^14^, ^15^ In our study, mean RMSV and LMSV values were found to be compatible with the literature. Also, no difference was found between the right and left sides for MSW, MSH, and MSL. This finding was consistent with the findings of the study of Al-Rawi et al.^7^ There are many studies investigating variations such as NSD and CB in the literature; some of them reported no significant relationship between the MSV and age,^7^, ^8^, ^12^, ^14^ while some others have found a significant relationship between age and MSV.^9^, ^10^ All patients included in the present study were selected from individuals aged 16 and over, since the osseous development of the sinuses would be completed in each patient, and the sinuses would have reached their adult sizes, not being affected by age variation. The present study showed a negative relationship between the LMSW and age, while other parameters and MSV values did not. Anbiaee et al.^9^ and Kalabalık et al.^10^ reported that MSV values decreased with age in their studies. According to our findings, the only parameter decreasing with age was the MSW. MSV and other parameters did not have a statistically significant relationship with the age variable, in accordance with the literature.^7^, ^8^, ^12^, ^14^

Al-Rawi et al.^7^ observed that the MSW, MSH, and MSL values were higher in males compared to females, and that there was a significant difference between genders. Our findings were consistent with those of Al-Rawi et al.^7^ in terms of sinus dimensions. However, in their studies, they reported that the MSV did not vary according to gender.^7^ Demir et al.^14^ also reported that MSV and gender types did not have a significant correlation. Nevertheless, many studies have reported that MSV values differ between genders and the MSV values of males are higher compared to females.^8^, ^9^, ^10^, ^15^ Our findings regarding the relationship between MSV and genders were consistent with the literature.^8^, ^9^, ^10^, ^15^

The prevalence of NSD, which is one of the most common variations of the nasal cavity, has been reported to range between 20% and 79%.^22^ According to our findings, NSD prevalence was observed at the rate of 66.7%. The incidence of the septal spur was 29.1%. While 27.5% of the patients with NSD had mild (G-1) deviation, 25% of them had moderate (G-2) and 14.2% of them had severe (G-3) deviation. It is known that severe NSD or presence of septal spur with NSD can narrow the meatus and prevent from air flow.^22^

In the present study, no significant correlation was found between NSD and MSV in both, ipsilateral and contralateral sides. Although there was no difference for MSV between the NSD subgroups, there were some differences between the subgroups in terms of dimensions. The mean LMSW value was significantly higher in the G-1 subgroup compared to G-2 and G-3 subgroups. The mean RMSW value was higher in the G-1 subgroup compared to the G-2. The relationships between the NSD and the ipsilateral or contralateral MSVs were found significant by several authors.^10^, ^12^, ^13^ However, Kalabalık et al.^10^ and Orhan et al.^12^ found the MSV on the ipsilateral side of NSD lower than the MSV on the contralateral side. Conversely, Gencer et al.^13^ observed that MSV on the ipsilateral side of the NSD side was higher compared to the contralateral side. Most of the researchers.^7^, ^8^, ^9^, ^15^ concluded that there is no significant relationship between NSD and MSV.

Kalabalık et al.^10^ investigated moderate and severe NSD subgroups and found significantly lower MSV values on the ipsilateral side of NSD, compared to the contralateral side in both groups. Karataş et al.^11^ divided patients into three groups, as mild, moderate, and severe according to the NSD severity, and found significant differences between the MSV values on the ipsilateral side of NSD and MSV values on the contralateral side for the moderate group. A statistically significant difference between the MSV values on the ipsilateral side of NSD of the mild and moderate groups, and moderate and severe groups was also reported in the abovementioned study.

Gencer et al.^13^ evaluated the subgroups based on the NSD severity and verified that the differences between the mild and severe groups and moderate and severe groups were significant while the difference between mild and moderate groups was insignificant for the MSV assessment. Moreover, they also reported lower MSV values on the ipsilateral side of NSD in severe NSD group, compared to other groups. Nonetheless, Al-Rawi et al.^7^ concluded that the severity of septal deviation had no effect on MSV. Our findings were compatible with the findings of Al-Rawi et al.

It has been reported that the incidence of middle concha pneumatization (CB), varies between 13% and 72.2%.^7^ In the present study, the incidence of CB was 62.5%. Some researchers reported that the relationship between CB and MSV was significant,^7^, ^15^ while some researchers could not find a significant relationship between them.^8^, ^9^, ^10^, ^14^ Demir et al.^14^ evaluated the effect of the CB subtypes on the sinus volume and found no significant correlation between them. Al-Rawi et al.^7^ and Kucybata et al.^15^ observed that both RMSV and LMSV values were significantly higher in patients with bilateral CB, compared to patients without CB or patients with unilateral CB.

However, Kucybata et al.^15^ highlighted that CB does not cause asymmetrical development of the maxillary sinus; explaining the differences in the bilateral CB group as an excessive growth of the sinus as the result of insufficient ventilation caused by bilateral CB. According to our findings, no significant difference was found between the presence of CB and MSV, and maxillary sinus dimensions. These results were compatible with the majority of the literature.

During an endoscopic surgery, the first procedure to reveal the maxillary sinus is generally the uncinectomy, therefore the uncinate process is an important anatomical landmark for the surgeons.^23^, ^24^ A contact of the pneumatized uncinate process with the ethmoid bulla or the middle concha may narrow the ethmoid infundibulum blocking the drainage of the maxillary sinus. UPP prevalence was 17.5% in the present study. There are no studies in the literature investigating the relationship between the uncinate process and MSV. Only in one study,^14^ the uncinate process angle was assessed. However, no significant relationship was found between the uncinate process angle and MSV. According to our findings, no significant difference was found regarding the effect of UPP on maxillary sinus dimensions and MSV.

RMSH of the group with unilateral right sided MCH was significantly higher compared to the group without MCH. MSW values of patients with bilateral ICH were lower compared to patients with unilateral left-sided ICH. Regarding the MSH and MSL, the values in the group with bilateral ICH were significantly lower compared to the group with unilateral ICH and group without ICH. However, both MCH and ICH had no effect on MSV. LMSW values were statistically higher in the group with unilateral right-sided PMC compared to the group without PMC. Although the presence of septa in the maxillary sinus is not associated with MSV; a notable increase in the LMSW and RMSW values was observed in the presence of septa in the maxillary sinus. There was no significant difference in MSH and MSL values related to the presence of septa. Our findings were consistent with the findings of Anbiaee et al.,^9^ they could not find a relationship between MSV and the presence of septa.

In the present study, the unilateral/bilateral and right/left distinctions of sinonasal anatomical variations were examined. Maxillary sinus measurements were recorded separately, not averaging the right and left sides. Therefore, an advantage of getting a better understanding of the differences between the measurements of the side with variations and the contralateral side was obtained. However, certain subgroups variations were not included in the statistical evaluation due to the insufficient number of patients. This fact should be considered as a limitation of the present study. The lack of evaluation of subgroups consisting of the combination of variations should also be mentioned as another limitation.

The maxillary sinus is the most relevant sinus to dentists due to its proximity to posterior maxillary teeth. In maxillofacial surgical procedures such as implant and orthognathic surgeries, the knowledge and detailed examination of the extension of the maxillary sinus is of great importance. In addition, CBCT provides very useful information regarding the possible relationship of the maxillary sinus with the nasal structures and three-dimensional radiological evaluation.

## Conclusion

The relationship between anatomical structures that form the nasal cavity and the maxillary sinus physiology is already well known. In the present study, the possible role of sinonasal region variations on maxillary sinus volume and dimensions was investigated. According to our results, there were significant relationships between genders and maxillary sinus dimensions and volume; NSD severity and MSW; MCH and MSH; ICH and some of the maxillary sinus dimensions; PMC and MSW; maxillary sinus septa and MSW, while there was no significant relationship between the evaluated anatomical variations and MSV. Comprehensive studies on this subject are lacking in the literature. Further studies involving a larger number of sample sizes should be conducted and the role of the combination of all variations on the sinus volume and dimensions should be evaluated separately.

## Funding

This research did not receive any specific grant from funding agencies in the public, commercial, or not-for-profit sectors.

## Conflicts of interest

The authors declare no conflicts of interest.
